# STING promotes senescence, apoptosis, and extracellular matrix degradation in osteoarthritis via the NF-κB signaling pathway

**DOI:** 10.1038/s41419-020-03341-9

**Published:** 2021-01-04

**Authors:** Qiang Guo, Ximiao Chen, Jiaoxiang Chen, Gang Zheng, Chenglong Xie, Hongqiang Wu, Zhimin Miao, Yan Lin, Xiangyang Wang, Weiyang Gao, Xiangtao Zheng, Zongyou Pan, Yifei Zhou, Yaosen Wu, Xiaolei Zhang

**Affiliations:** 1 Department of Orthopedics, The Second Affiliated Hospital and Yuying Children’s Hospital of Wenzhou Medical University, Wenzhou, 325000 Zhejiang Province China; 2 Key Laboratory of Orthopaedics of Zhejiang Province, Wenzhou, 325000 Zhejiang Province China; 3 The Second School of Medicine, Wenzhou Medical University, Wenzhou, 325000 Zhejiang Province China; 4 Department of Orthopedics, Affiliated Hospital of Guilin Medical College, Guilin, 541000 Guangxi Province China; 5 Department of Vascular Surgery, The Second Affiliated Hospital and Yuying Children’s Hospital of Wenzhou Medical University, Wenzhou, 325000 Zhejiang Province China; 6 Department of Orthopaedics, The Second Affiliated Hospital of Zhejiang University School of Medicine, Hangzhou, 310000 Zhejiang Province China; 7 Chinese Orthopedic Regenerative Medicine Society, Hangzhou, 310000 Zhejiang Province China

**Keywords:** Apoptosis, Senescence

## Abstract

Damaged deoxyribonucleic acid (DNA) is a primary pathologic factor for osteoarthritis (OA); however, the mechanism by which DNA damage drives OA is unclear. Previous research demonstrated that the cyclic GMP-AMP synthase (cGAS)-stimulator of interferon genes (STING) participates in DNA damage response. As a result, the current study aimed at exploring the role STING, which is the major effector in the cGAS-STING signaling casacde, in OA progress in vitro, as well as in vivo. In this study, the expression of STING was evaluated in the human and mouse OA tissues, and in chondrocytes exposed to interleukin-1 beta (IL-1β). The influences of STING on the metabolism of the extracellular matrix (ECM), apoptosis, and senescence, were assessed in STING overexpressing and knocking-down chondrocytes. Moreover, the NF-κB-signaling casacde and its role in the regulatory effects of STING on ECM metabolism, apoptosis, and senescence were explored. The STING knockdown lentivirus was intra-articularly injected to evaluate its therapeutic impact on OA in mice in vivo. The results showed that the expression of STING was remarkably elevated in the human and mouse OA tissues and in chondrocytes exposed to IL-1β. Overexpression of STING promoted the expression of MMP13, as well as ADAMTS5, but suppressed the expression of Aggrecan, as well as Collagen II; it also enhanced apoptosis and senescence in chondrocytes exposed to and those untreated with IL-1β. The mechanistic study showed that STING activated NF-κB signaling cascade, whereas the blockage of NF-κB signaling attenuated STING-induced apoptosis and senescence, and ameliorated STING-induced ECM metabolism imbalance. In in vivo study, it was demonstrated that STING knockdown alleviated destabilization of the medial meniscus-induced OA development in mice. In conclusion, STING promotes OA by activating the NF-κB signaling cascade, whereas suppression of STING may provide a novel approach for OA therapy.

## Introduction

Osteoarthritis (OA) is a degenerative disease that may cause disability for individuals and substantial burden for the society^1^. The disease has a complex etiology; aging, obesity, metabolic abnormalities, osteoporosis, joint deformity, and other factors are all implicated in OA pathogenesis^2^. Although OA has multiple pathological changes, structural destruction, and dysfunction of the articular cartilage are the hallmark features of OA pathology^3,4^. The chondrocyte is the only type of cell that resides in the articular cartilage, and its abnormal status (apoptosis and senescence) and function (extracellular matrix (ECM) synthesis and degradation) may lead to OA progression in affected subjects^5^.

For a long time, deoxyribonucleic acid (DNA) damage has been associated with the pathogenesis of various diseases^6,7^. Previous studies have indicated that DNA damage drives the progression of OA. Moreover, research has shown that damages on genomic and mitochondrial DNA are substantially increased in the course of OA pathology^8^. Further, research shows that pathological factors of OA, such as reactive oxygen and nitrogen species (ROS and RNS) and inflammatory cytokines (IL-1β and TNF-α), may induce DNA damage in vitro^9^. Oscar et al. reported that the expression of REDD1, which is responsible for cellular DNA damage response, was reduced during ageing and OA. Furthermore, DNA damage is associated with apoptosis and senescence in chondrocytes^10^. These findings suggest that DNA damage is vital for the pathogenesis of OA. However, it is still not clear how DNA damage causes abnormal status and function in chondrocytes.

Under physiological conditions, DNA mainly resides in the nucleus and mitochondria of cells^11^. When DNA damage occurs, DNA may leak into the cytoplasm. The cyclic GMP-AMP synthase (cGAS)-stimulator of Interferon genes (STING) is an innate immune pathway whose primary function is to recognize, as well as respond to cytosolic DNA. With cytosolic DNA presence, cGAS recognizes and binds to the cytoplasmic DNA and recruits STING to initiate a series of downstream reactions^12^. Research evidence has chronicled that cGAS-STING cascade activation may cause various pathological conditions^13,14^. Jauhari et al.^15^ reported that melatonin deficiency causes mitochondrial DNA release, which further activates the cGAS-STING pathway and its downstream interferon regulatory factor 3 signaling. Moreover, it stimulates the production of inflammatory cytokines, thereby accelerating ageing and neurodegeneration. Luo et al. documented that cGAS-STING cascade could be activated by cytoplasmic chromatin fragments, which cause senescence-associated secretory phenotype (SASP) and accelerates senescence^16^. Tang et al.^17^ demonstrated that the activation of STING triggers apoptosis in malignant B Cells. These findings suggest that cGAS-STING is linked with inflammation, senescence and apoptosis; however, its role in OA is unclear.

Herein, we evaluated the expression of STING, which is the major effector of cGAS-STING signaling pathway, in OA progress; STING was also overexpressed and knocked down to assess its effects on senescence, apoptosis and ECM metabolism in chondrocytes; STING knockdown lentivirus was intra-articularly injected to evaluate the therapeutic effect of STING suppression on OA progress. We found that STING may promote senescence, apoptosis, and cause ECM metabolism imbalance in chondrocytes, whereas suppression of STING may provide a new approach for OA therapy.

## Results

### The expression of STING is increased in osteoarthritic human and mouse articular cartilage

**Fig. 1:**
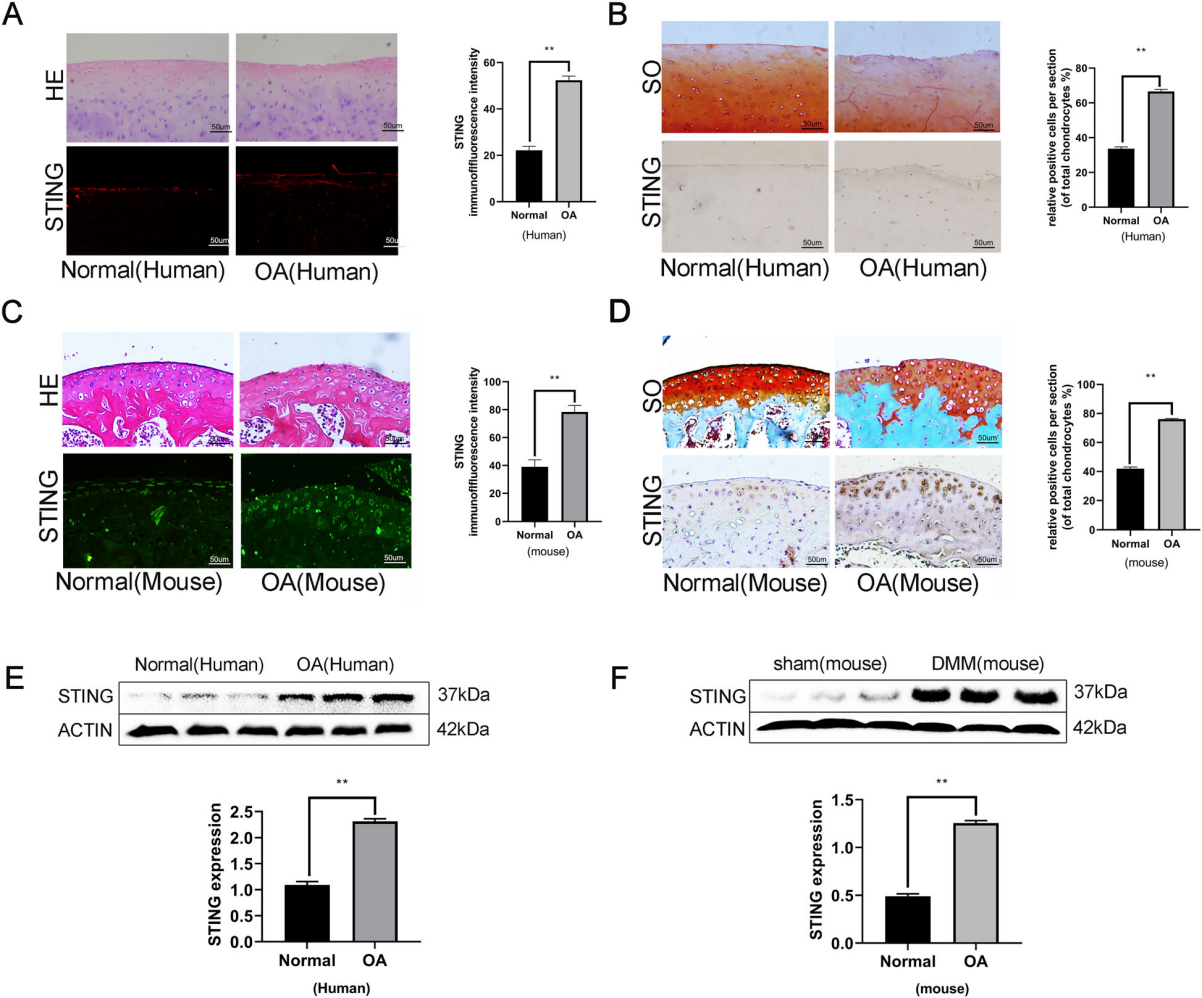
The expression of STING is increased in osteoarthritic human and mouse articular cartilage. **A**–**B** Representative H&E staining, Safranin O staining as well as immunofluorescence staining, immunohistochemistry staining in human articular cartilage from normal, as well as OA patients (bar: 50 μm). **C**–**D** Representative H&E staining, Safranin O staining, as well as immunofluorescence staining, immunohistochemical staining in mouse knee articular cartilage from sham, as well as DMM models (bar: 50 μm). **E** The protein expression of STING in chondrocytes originated from normal and degenerated specimens of human. **F** The protein expression of STING in chondrocytes originated from normal group and DMM (degenerated) specimens of mouse. All data are indicated as the mean ± SD (*n* = 5); **P < 0.01.

To determine STING’s role in OA development, healthy and osteoarthritic tissues of human as well as mouse articular cartilages were collected. We compared STING expression levels in the normal, as well as OA articular cartilage via immunofluorescence and immunohistochemistry (Fig. 1A, B). We found that the expression level of STING was increased in OA patients in contrast with the expression levels in normal patients. Similarly, the STING expression level was remarkably increased in the OA articular cartilage of the destabilization of the medial meniscus (DMM)-induced mouse model in contrast with the normal control (Fig. 1C, D). Besides, we isolated chondrocytes from the normal and OA patients for western blot assessment. As indicated in Fig. 1E, the STING expression was increased in OA patient-derived chondrocytes compared with the STING’s expression in the healthy patient-derived chondrocytes. Western blot analysis of the isolated cartilage tissues showed that STING expression was remarkably increased in the DMM-triggered OA mice in contrast with in the normal control (Fig. 1F). Thus, it was concluded that in OA, the level of STING was increased.

### The expression of STING is elevated in IL-1β-treated chondrocytes

**Fig. 2:**
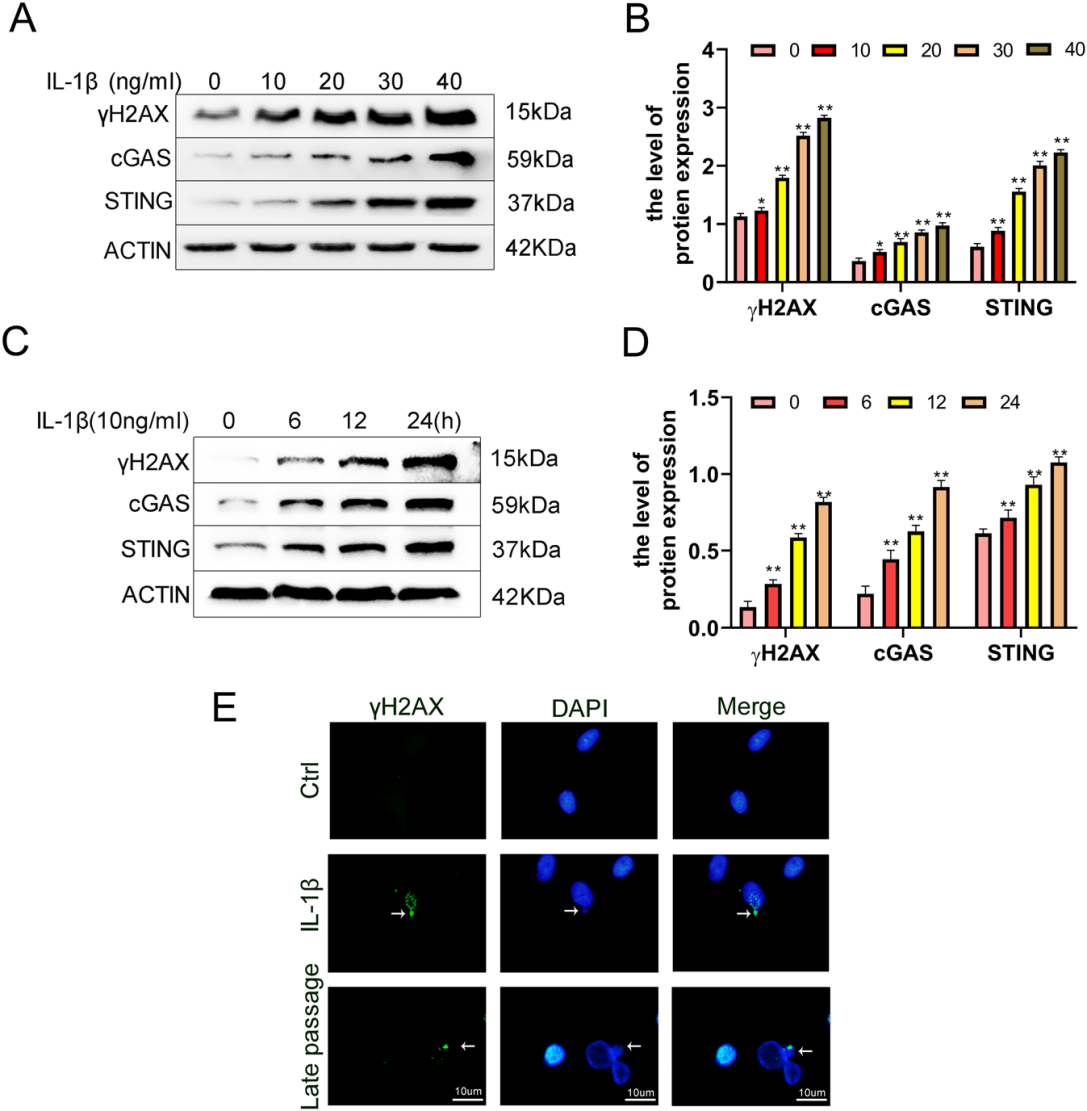
The expression of STING is increased in IL-1β-triggered chondrocytes. The protein expression of γh2ax, cGAS, STING in chondrocytes were explored by western blot and its quantification was detected by ImageJ. **A**–**B** The impact of different concentrations of treatment with IL-1β on mouse chondrocytes. The chondrocytes were exposed to 0, 10, 20, 30, 40 (ng/ml) IL-1β for 24 h. **C**–**D** The impact of different durations of treatment with IL-1β on mouse chondrocytes. The chondrocytes were incubated with IL-1β (10 ng/ml) for 0, 6, 12, 24 h. **E** Representative immunofluorescence staining of γh2ax in IL-1β (10 ng/ml, 24 h) induced chondrocytes (bar:10μm). All data were indicated as the mean ± SD (*n* = 5); *P < 0.05, **P < 0.01.

Pro-inflammatory cytokines such as the IL-1β are closely linked with OA occurrence. Thus, we utilized IL-1β to mimic OA in vitro. Western blot evaluation demonstrated that exposure to IL-1β upregulated the quantities of γh2ax (DNA damage marker) and cGAS-STING in chondrocytes in a time, as well as dose-dependent approach (Fig. 2A–D). Besides, immunofluorescence analysis showed that γh2ax expression was remarkably elevated in IL-1β-treated and the passage-triggered senescent chondrocytes. Furthermore, the presence of damaged DNA was observed outside the nucleus (Fig. 2E). These results were consistent with studies in vivo, indicating that the expression of STING was upregulated during OA process.

### STING induces ECM degradation and promotes senescence and apoptosis in chondrocytes

**Fig. 3:**
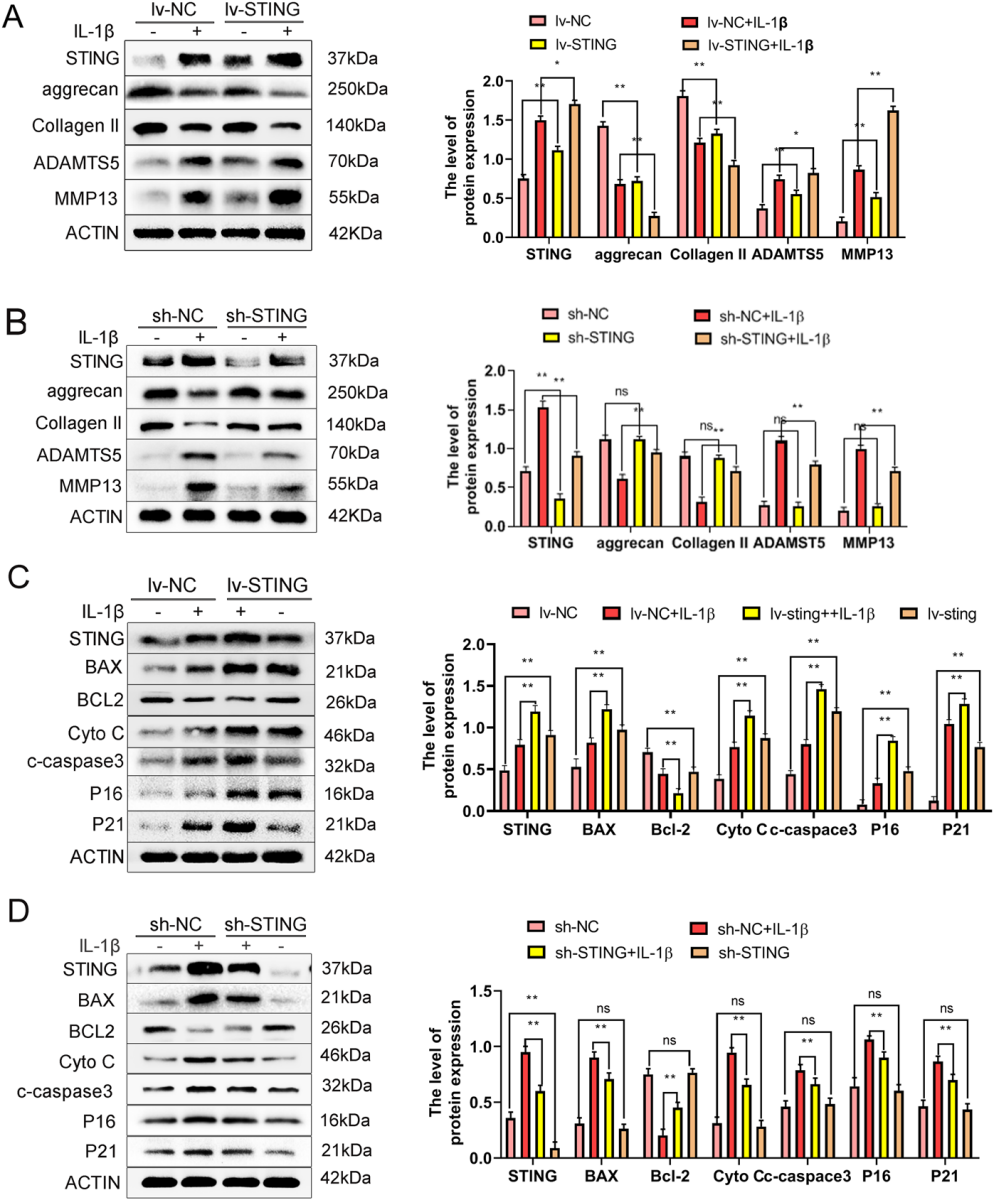
STING promotes ECM degradation, senescence, and apoptosis in chondrocytes. The mouse chondrocytes were pre-exposed to lv-STING or sh-STING and subsequently by IL-1β (10 ng/ml) treatment for 24 h. **A** Western blot, as well as its quantification revealed the level of STING, Aggrecan, and Collagen II, MMP13 and ADAMTS5 after exposure to IL-1β (10 ng/ml) or lv-STING. **B** Western blot along with its quantification revealed the level of STING Aggrecan, and Collagen II, MMP13 and ADAMTS5 after treatment with IL-1β or sh-STING. **C** Western blot along with its quantification revealed the level of STING, BAX and BCL-2, and Cyto-c, cleaved-caspase-3 and p16INK4a, and p21 after exposure to IL-1β or lv-STING. **D** Western blot along with its quantification revealed the level of STING, BAX, and BCL-2, and cleaved-caspase-3 and p16INK4a, and p21 after exposure to IL-1β or sh-STING. All data are indicated as mean ± SD (*n* = 5); *P < 0.05, **P < 0.01.

OA is closely correlated with the degradation of the ECM. Therefore, we explored the ECM-related proteins (Collagen II, ADAMTS5, Aggrecan, as well as MMP13) via the western blotting assay. The lenti-STING (lv-STING) and lenti-sh-STING (sh-STING) were used for STING overexpression and knockdown, respectively, in chondrocytes. The results demonstrated that STING overexpression increased the expression of ADAMTS5, MMP13, and decreased Collagen II as well as Aggrecan expression. These findings were consistent with those of IL-1β treatment (Fig. 3A). Conversely, the IL-1β-stimulated expression of ADAMTS5, MMP13, collagen II, and Aggrecan stimulated was reversed by sh-STING; while STING knockdown alone did not affect the expression level of these proteins (Fig. 3B).

OA is also correlated with senescence and apoptosis of chondrocyte. In light of this, we evaluated the role of STING overexpression and knockdown in senescence and apoptosis in chondrocytes. The level of cellular senescence was estimated by analyzing the expression quantities of p16INK4a and p21 in the chondrocytes. The results showed higher p16INK4a and p21 protein measures in the STING-overexpressed and IL-1β-treated chondrocytes (Fig. 3C). Although STING knockdown significantly reduced the IL-1β-simulated upregulation of p16INK4a and p21 proteins, the knockdown of STING alone did not affect these proteins’ expression levels (Fig. 3D). To examine the effects of STING on apoptosis, the quantities of BAX, cleaved-caspase-3, BCL-2, as well as cytochrome c (Cyto-c) were explored. We found that the overexpression of STING upregulated the expression levels of the apoptosis-associated proteins (BAX, Cyto-c, as well as cleaved-caspase-3) levels and downregulated the expression of BCL-2 quantities (Fig. 3C); knockdown of STING significantly reversed the effect of IL-1β simulation, and STING knockdown alone did not alter the expression levels of BAX, cleaved-caspase-3, BCL-2, as well as Cyto-c expression quantities (Fig. 3D). In conclusion, STING induces ECM degradation and promotes apoptosis and senescence in chondrocytes.

### STING induces ECM degradation through the NF-κB-signaling axis

**Fig. 4:**
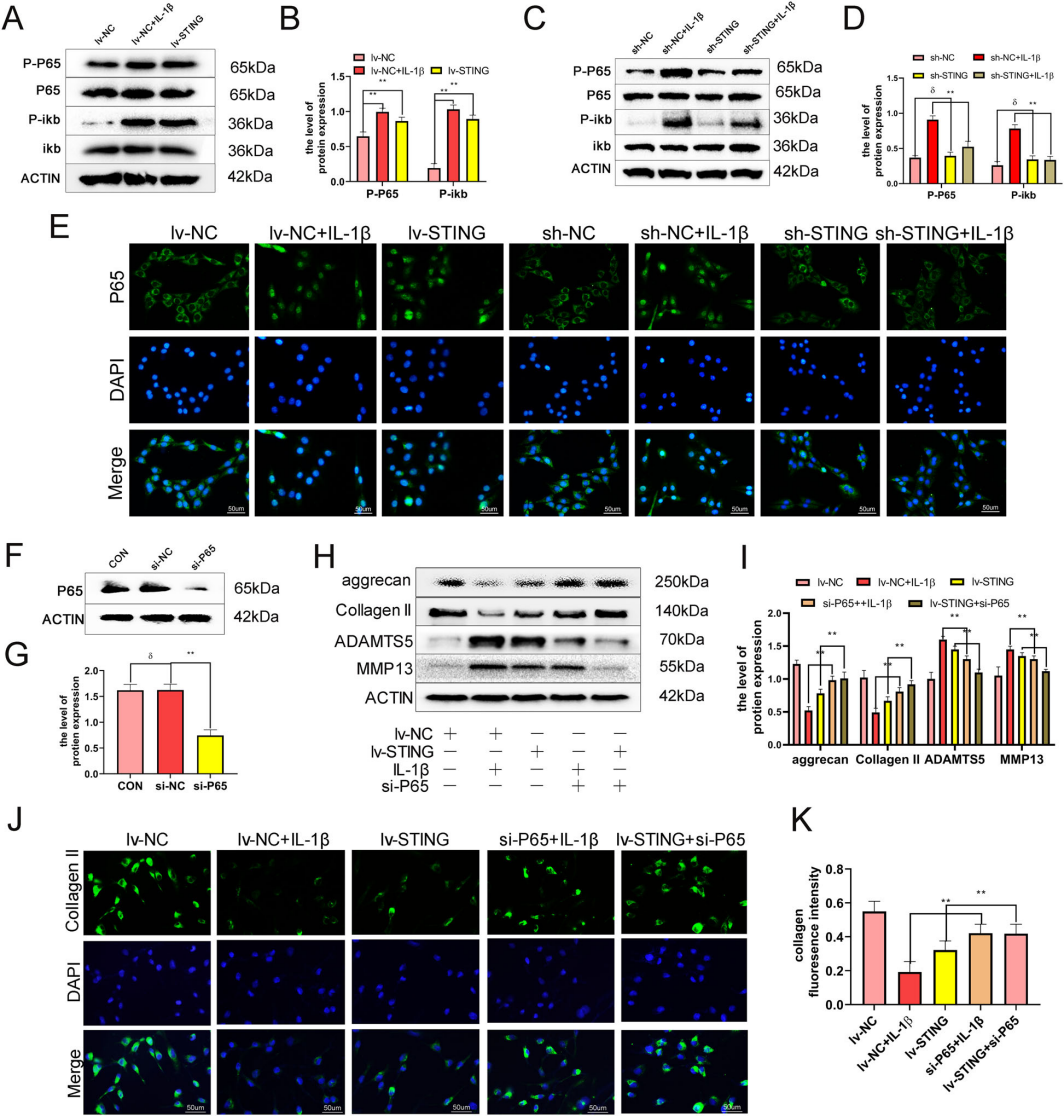
STING induces ECM degradation via NF-κB activation. The mouse chondrocytes were pre-exposed to lv-STING or sh-STING or si-P65 followed by exposure to IL-1β (10 ng/ml) for 24 h. **A**–**B** Western blot as well as its quantification revealed the phosphorylation level of P65 and iκb after exposure to IL-1β or lv-STING. **C**–**D** Western blot along with its quantification revealed the phosphorylation level P65, as well as iκb after exposure to IL-1β or sh-STING. **E** Immunofluorescence staining of P65 in chondrocytes (bar: 50 μm). **F**–**G** Western blot along with its quantification revealed that the siRNA-mediated P65 knockdown was successful. **H**–**I** Western blot along with its quantification revealed that the level of Collagen II, Aggrecan, MMP13, and ADAMTS5 as treated above. **J**–**K** Immunofluorescence staining of collagen II in chondrocytes as treated above (bar:50μm). All data were presented as mean ± SD (*n* = 5); **P < 0.01.

The ECM’s degradation is closely associated with chondrocyte inflammation, which is regulated via the NF-κB-signaling pathway^18^. Recent studies have showed that NF-κB acts as a downstream effector of STING and is involved in the STING-mediated-inflammation^19^. In order to investigate whether STING induces the degradation of the ECM through the NF-κB-signaling casacde, lv-STING was used to upregulate STING protein in chondrocytes. The results showed that higher phosphorylation of P65 and iκb protein levels were seen in STING overexpressing and IL-1β treated chondrocytes (Fig. 4A, B), whereas knockdown of STING significantly reduced IL-1β-simulated upregulation of P65 and iκb protein phosphorylations (Fig. 4C, D). Furthermore, the analysis of the immunofluorescence of nuclear P65 confirmed that STING activates NF-κB signaling (Fig. 4E). To further explore the NF-κB role in the STING-mediated modulation of the ECM, si-P65 was used to downregulate P65 protein in chondrocytes (Fig. 4F, G). We found that the addition of si-P65 significantly reverse the Lv-STING-mediated downregulation in the expression of collagen II, as well as Aggrecan and upregulation of ADAMTS5 and MMP13 (Fig. 4H, I). Analysis of collagen II immunofluorescence further confirmed that STING mediates the degradation of the ECM degradation through the NF-κB-signaling cascade (Fig. 4J, K). In conclusion, STING induces the degradation of the ECM degradation through the NF-κB-signaling pathway.

### STING regulates senescence and apoptosis of chondrocyte via NF-κB-signaling pathway

**Fig. 5:**
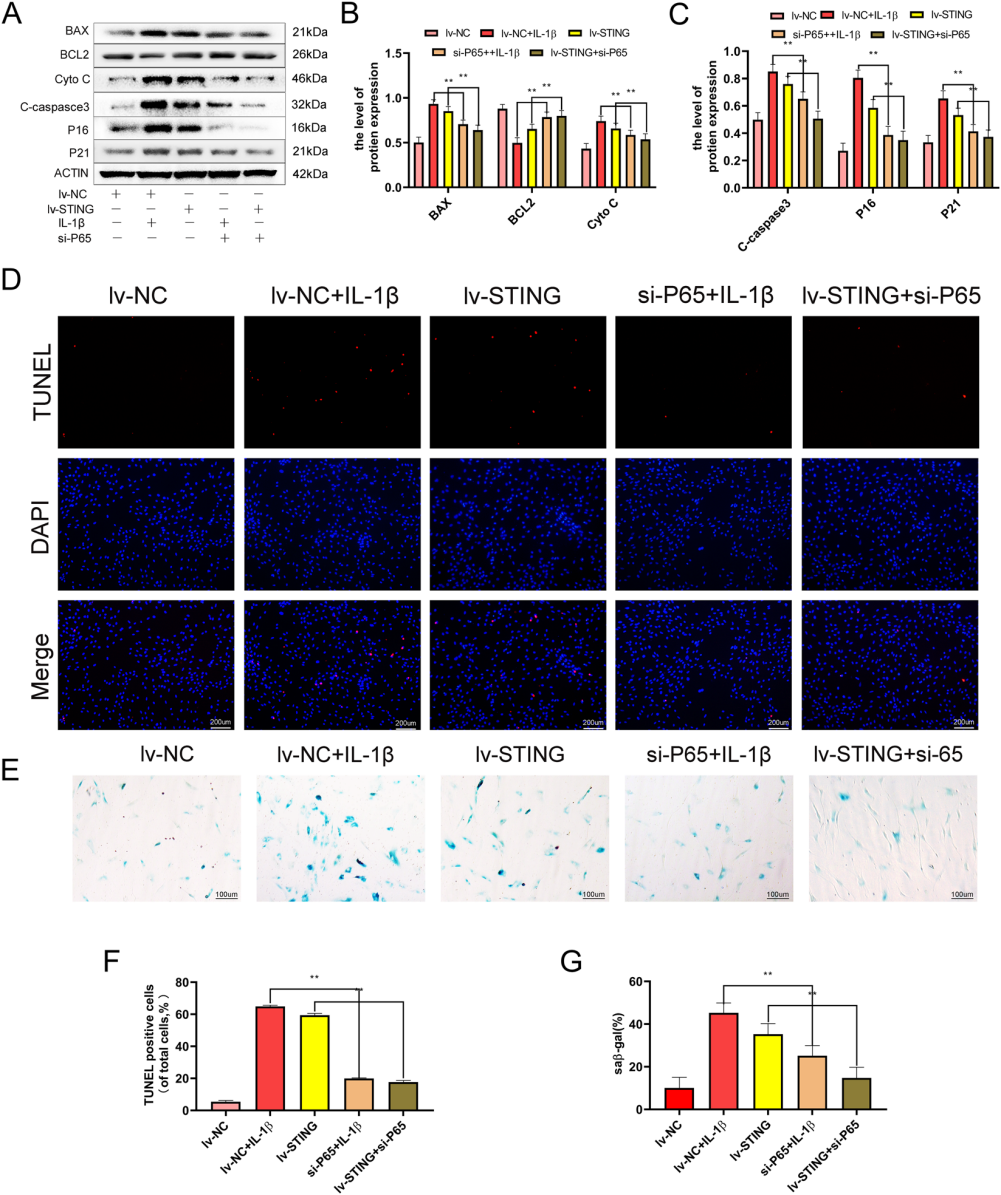
STING induces chondrocyte senescence and apoptosis via NF-κB activation. The mouse chondrocytes were pre-exposed to lv-STING or si-p65 followed by exposure to IL-1β (10 ng/ml) for 24 h. **A**–**C** Western blot and its quantification of the level of BAX, BCL-2, Cyto-c, cleaved-caspase-3, p16INK4a and p21. **D**–**F** TUNEL staining assay was conducted on the chondrocytes, as treated above (bar: 100 μm). **E**–**G** SA-β-gal staining assay was carried out on chondrocytes, as shown (bar: 50 μm). All data were indicated as mean ± SD (*n* = 5); **P < 0.01.

Apoptosis and senescence are the main pathological characteristics of OA^20,21^. Previous research demonstrate that the NF-κB-signaling pathway participates in senescence and apoptosis^22^. Thus, we explored the role of the NF-κB signaling role in the STING-induced apoptosis and senescence in chondrocytes. We established that si-P65 remarkably reduced the expression of STING-induced senescence-associated (p16INK4a and p21), and apoptosis-associated (BAX, Cyto-c, as well as cleaved-caspase-3) proteins and increased the expression of BCL-2 (Fig. 5A–C). Further, the SA-β-gal and TUNEL staining confirmed that STING regulates senescence and apoptosis via the NF-κB-signaling axis activation (Fig. 5D–G). In summary, the results revealed that si-p65 reverses STING-induced senescence and apoptosis in chondrocytes, and STING regulates senescence and apoptosis of chondrocytes through the NF-κB-signaling casacde.

### STING knockdown ameliorates OA in mice

**Fig. 6:**
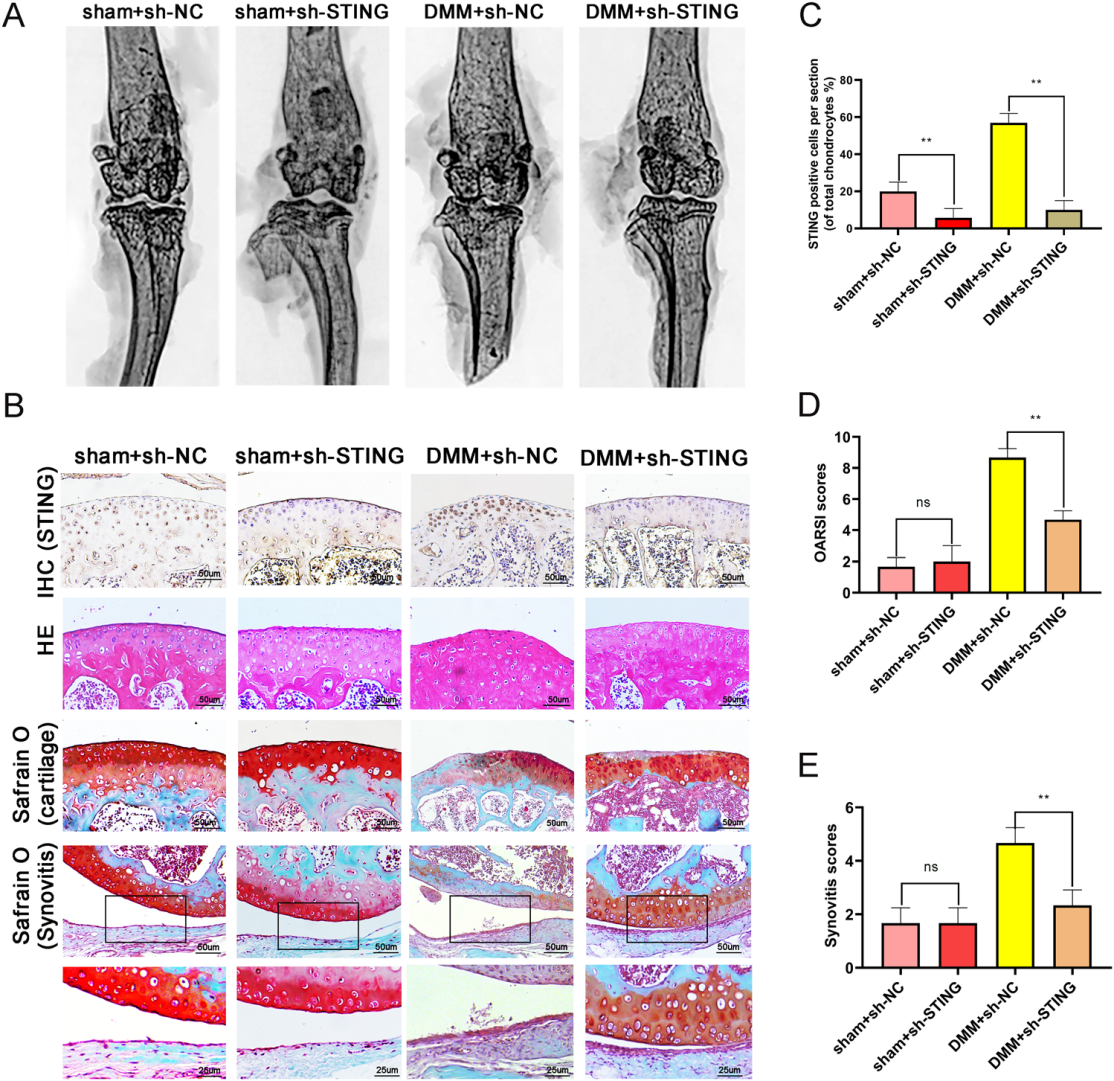
STING knockdown ameliorates osteoarthritis in vivo. The osteoarthritis mouse model was created by surgical destabilization of the medial meniscus (DMM), after 8-week time period, the pathology of OA was assessed by X-ray or stained with H&E, as well as Safranin O. **A** Digital X-ray image of mouse knee joints from different experimental groups. **B** Representative immunohistochemistry of STING, S-O staining of the cartilage and synovitis in the four groups at eight weeks post-surgery (bar: 50 or 25 μm). **C** Quantitative analysis of immunohistochemistry of STING was detected by image J. **D**–**E** The OARIS scores of the cartilage and the scores of the synovitis in the four groups. All data were presented as mean ± SD (*n* = 15), **P < 0.01.

**Fig. 7:**
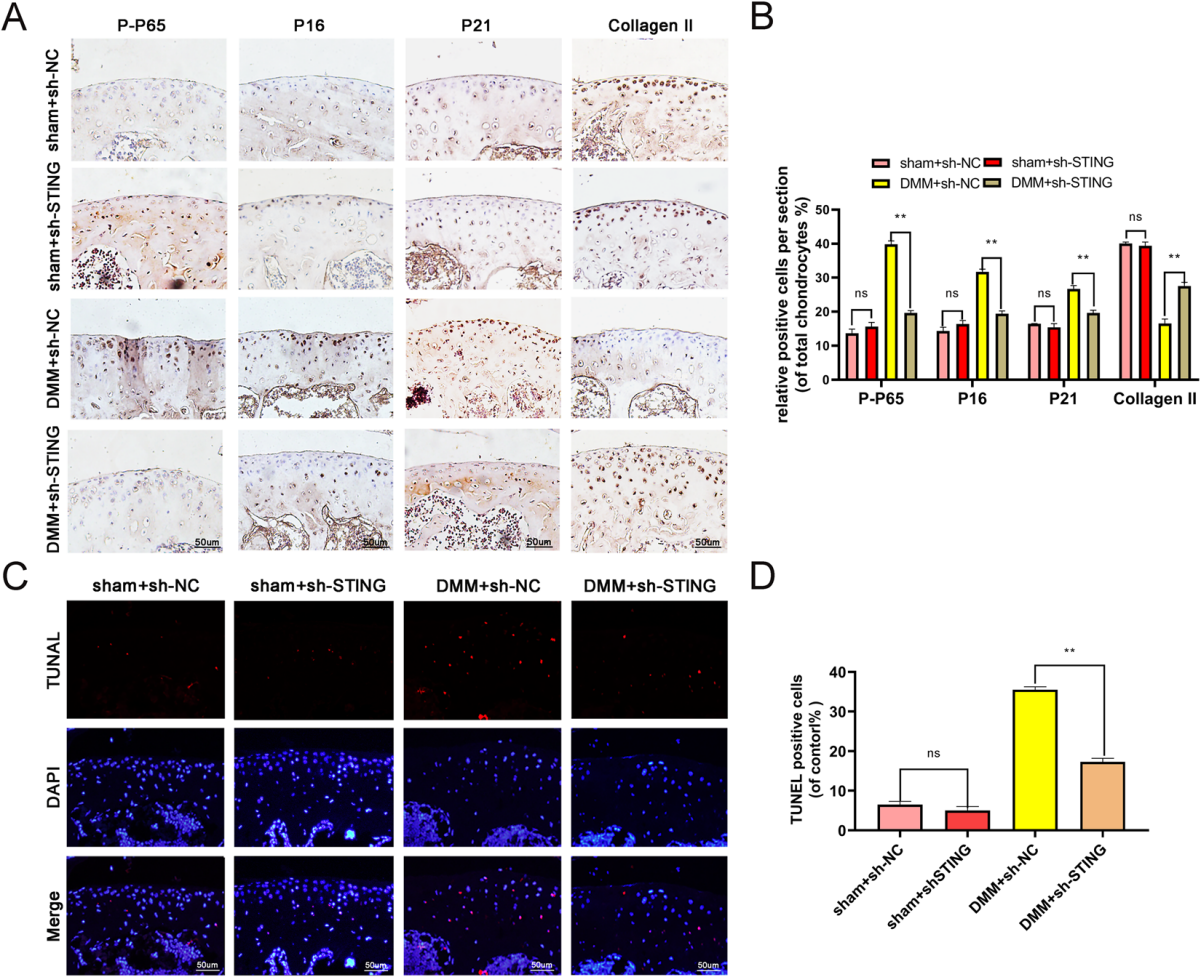
STING knockdown ameliorates the degradation of the ECM, senescence, and apoptosis by inhibiting NF-κB in vivo. **A**–**B** The expression of p-P65, p16INK4a, P21, and collagen II were evaluated by immunohistochemistry in mouse cartilage (bar: 50 μm). **C–D** TUNEL staining assay in mouse cartilage (bar: 50 μm). All data were presented as mean ± SD (*n* = 15); **P < 0.01.

In vivo therapeutic effects of STING knockdown were evaluated by injecting lentiviral particles sh-STING into the knee cavity of mice, after which X-ray imaging as well as Safranin O staining were performed to evaluate the histomorphological differences. Immunohistochemistry results showed that successful lentivirus-mediated STING knockdown at two weeks post lentivirus injection (Fig. 6B). The postoperative joint space was abnormally narrow, and the density of the cartilage surface was increased in DMM + sh-NC group compared to sham + sh-NC group, indicating successful establishment of OA model in mice. The DMM + sh-STING group had less calcification on the cartilage surface and a lower joint space stenosis than the DMM + sh-NC group (Fig. 6A). Safranin O staining revealed erosion on the articular cartilage surfaces, decreased number of cells, synovial proteoglycan loss, synovial thickening, and synovial hyperplasia in DMM + sh-NC group compared with the DMM + sh-STING group after 2 months following surgery. The DMM + sh-STING group had a relative complete cartilage surface, more proteoglycan, as well as thinner synovium compared with the DMM + sh-NC group (Fig. 6B). Also, the Osteoarthritis Research Society International (OARSI) and synovitis scores were remarkably lower in the DMM + sh-STING group in contrast with the DMM + sh-NC group (Fig. 6D, E). Immunohistochemical staining and quantitative results revealed that knockdown of STING reduced the levels of p-P65, p21, MMP13, ADAMTS5, and p16INK4a proteins, and promoted the levels of Collagen II protein in chondrocytes. However, no remarkable difference was reported in the level of total P65 in each group (Fig. 7A, B and Fig. S1 A, B). In addition, tissue TUNEL staining confirmed that STING knockdown decreases the level of chondrocyte apoptosis (Fig. 7C, D). These results suggest that STING knockdown ameliorates OA in mice.

## Discussion

DNA damage causes an imbalance in cellular homeostasis, which leads to inflammation, apoptosis, and senescence^23,24^. OA is an age-related chronic degenerative disease. Growing evidence shows a close association between age-related DNA damage and the incidence of OA^25,26^. Therefore, understanding the cellular response to DNA damage in chondrocytes is crucial to exploring OA’s pathogenesis in affected patients.

In physiologic states, the status of cellular DNA is tightly monitored. When DNA damage occurs, it is repaired endogenously; however, when the repair rate is slower than the damage rate, the damaged DNA accumulates and exits the nucleus or mitochondria to become cytosolic DNA^27,28^. The cGAS-STING signaling is a newly discovered cascade that may respond to cytosolic DNA. Previous reports indicate that cytosolic DNA-induced cGAS-STING activation promotes the production of inflammatory cytokines in inflammatory autoimmune diseases^29^. Inflammatory cytokines, such as the IL-1β, involved in OA progression, have been documented to activate the cGAS-STING signaling casacde^30^. This implies that the cGAS-STING cascade may be linked with the pathogenesis of OA.

Activation of the cGAS-STING pathway has been demonstrated to induce senescence. Yang et al. reported that cGAS knockout increases proliferation, decreases SA-β-gal-positive cells, and SASP in mouse embryonic fibroblasts^31^, which has also been confirmed by Gluck’s study^32^. The deficiency of STING may also lead to decreased SASP in vivo^33^. However, these findings were based mainly on fibroblasts, whether the cGAS-STING pathway may regulate senescence in other cells is still unclear.

We explored the function of STING on chondrocyte senescence in OA. In Herein, we found that STING’s expression level in the osteoarthritic articular cartilage tissues and IL-1β induced osteoarthritic chondrocytes was high. Furthermore, IL-1β may time-, as well as dose-dependently trigger increased DNA damage and STING expression in a time and dose-dependent manner. Similar to the existing results, we found in chondrocytes that knockdown of STING may cause decreased senescence; we also found that overexpression of STING may lead to increased senescence, which has not been reported before.

Knockdown of cGAS and STING may induce decreased phosphorylation of NF-κB p65/RelA subunit in human lung fibroblast IMR90 cells, indicating cGAS-STING may regulate NF-κB signaling^33^; whereas NF-κB signaling is related to SASP and involved in senescence. Through the overexpression of STING and the inhibition of NF-κB signaling, we provide evidence that cGAS-STING may indeed regulate NF-κB signaling in chondrocytes, and the mechanism that STING promotes senescence in chondrocytes is through activation of NF-κB signaling.

The cGAS-STING pathway has been demonstrated to regulate apoptosis. Gulen et al. reported that small-molecule STING agonist 10-carboxymethyl-9-acridanone may induce apoptosis in CD4+ T cells. Nonetheless, the apoptotic effect of STING was shown to be cell type-dependent, as it may not induce apoptosis in bone mouse marrow-derived macrophages, bone marrow-derived DCs, as well as primary embryonic fibroblasts^34^. Here, we demonstrated that STING overexpression promotes the expression levels of BAX, Cyto-c, as well as cleaved-caspase-3 and decreases the expression levels of BCL-2, suggesting that STING induces apoptosis in chondrocytes. Meanwhile, STING knockdown may alleviate IL-1β induced apoptosis, indicating STING could regulate apoptosis in various conditions (at least in IL-1β inflammatory condition).

Besides senescence and apoptosis, impaired ECM metabolism also contributes to OA^35^. Research has revealed that the cGAS-STING pathway may regulate senescence and apoptosis; however, its role in regulating the ECM metabolism regulation has never been investigated. For the first time, we report that STING may regulate the metabolism of ECM in chondrocytes. This study shows that STING overexpression increases the levels of ADTAMTS5, as well as MMP13 and reduces the levels of type II Collagen, as well as Aggrecan, whereas its knockdown causes the opposite effect. The mechanism by which STING regulates ECM metabolism is related to the NF-κB signaling cascade. Blockage of the NF-κB signaling axis by si-P65 attenuated the STING overexpression-induced ECM metabolism, suggesting that STING may regulate ECM metabolism through the NF-κB signaling cascade.

**Fig. 8:**
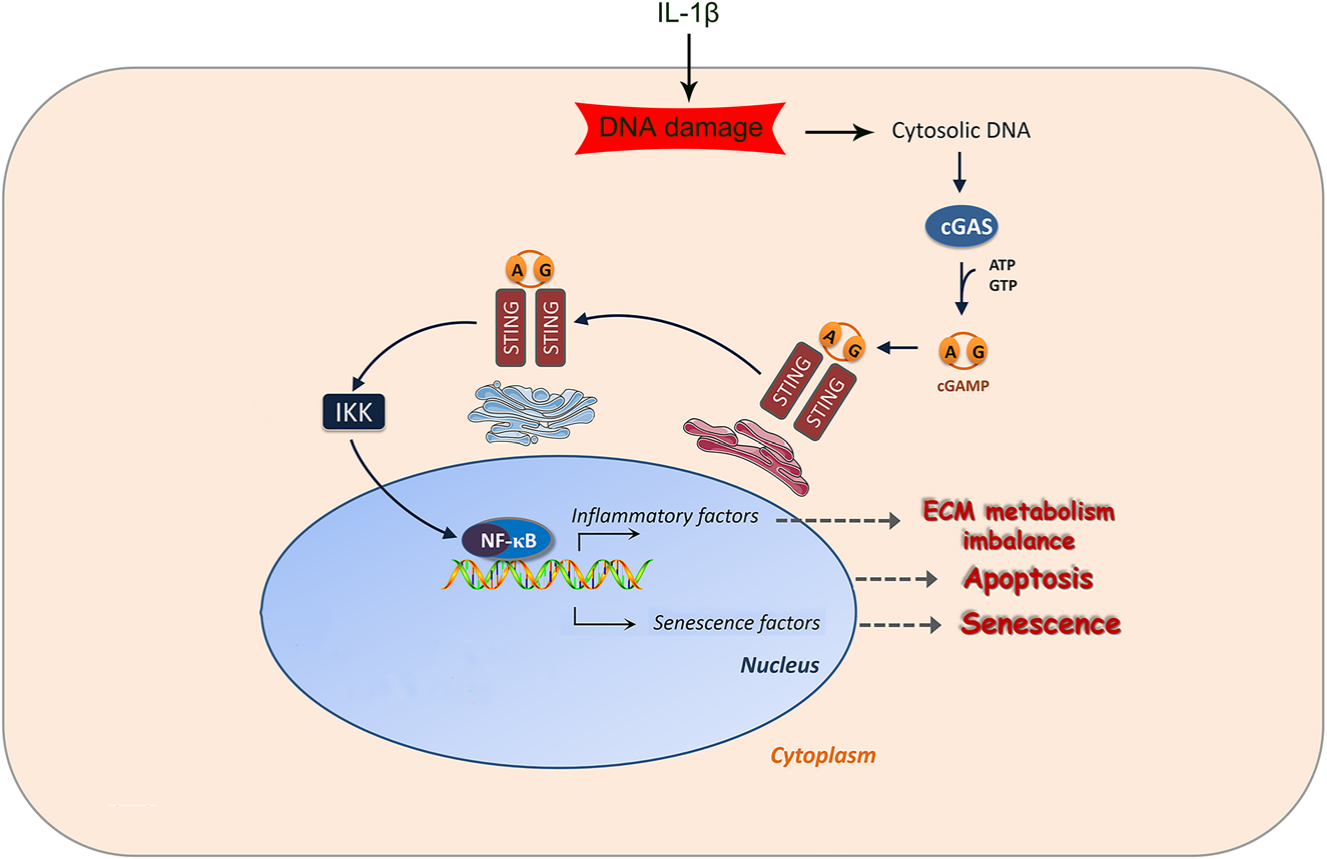
Schematic illustration of effect of cGAS-STING in osteoarthritis development.

NF-κB is a crucial signaling pathway that responds to inflammatory cytokines in chondrocytes^36,37^. Previous studies demonstrated that inflammatory cytokines, e.g., IL-1β, may activate the NF-κB signaling pathway and cause downstream effects such as apoptosis and ECM metabolism imbalance^38,39^. Our study suggests that inflammatory cytokines, especially IL-1β, may also activate NF-κB-signaling pathway through an indirect way. Inflammatory cytokines may induce DNA damage in cells, while cytosolic DNA may activate cGAS-STING pathway and further cause NF-κB signaling pathway activation (Fig. 8).

Therefore, either the NF-κB signaling or the cGAS-STING pathway or both can be targeted to treat inflammation-induced OA. Lots of compounds have been documented to be potent NF-κB-signaling pathway inhibitors^40,41^, and many of them have been shown to have therapeutic effects on OA^42^. Besides NF-κB signaling inhibitors, cGAS-STING pathway inhibitors have also been developed. Haag et al.^43^ performed a cell-based chemical study on IFNβ activity and uncovered two nitrofuran derivatives (C-178 as well as C-176) that may strongly reduce STING activity; however, whether these STING antagonists may alleviate OA pathology requires further verification. Meanwhile, the cGAS antagonists should also be tested in OA therapy^44^.

Besides the NF-κB- and cGAS-STING-signaling pathways, which are the effectors of DNA damage, we could also target DNA damage inducers in OA. DNA damage could be caused by numerous endogenous or exogenous stresses, including oxidative stress, oncogenic mutations, genotoxic stress, telomere erosion, as well as metabolic stress^45^. Therefore, strategies should be developed to target endogenous or exogenous stresses. Downregulation of cytoplasmic DNases may be responsible for the cytoplasmic accumulation of DNA^46^; thus, they should also be considered as potential targets for OA therapy.

## Materials and methods

### Reagents and antibodies

Recombinant rat IL-1β was acquired from Peprotech (RockyHill, NJ, USA). Abcam (Cambridge, MA, USA) provided the antibodies against yH2ax (ab81299), cGAS (ab179785), STING (ab179775), MMP13 (ab39012), ADAMTS5 (ab41037), Collagen II (ab239007), Aggrecan (ab3778), p21 (ab107099) and p16 (ab51243). Moreover, the Cell Signaling Technology (Beverly, MA, USA) provided the antibodies against β-actin (#3700).

### Human and mouse cartilage samples collection and assessment

Five OA patients’ articular cartilage specimens were obtained from total knee arthroplasty (aged 48–67; mean, 59.6 years; Kellgren–Lawrence grade III or IV). Five healthy cartilage specimens were obtained from trauma patients without OA and no obvious clinical, as well as imaging features of OA (aged 45–65; mean, 57.2 years; Kellgren–Lawrence grade 0 or I). Five cases of mouse OA articular cartilage specimens were obtained from DMM model. Five healthy cartilage specimens were obtained from sham group. The cartilages were sectioned into 5 mm sagittal segments and embedded-embedded for histological analysis.

Chondrocytes from these tissues were also cultured to evaluate the expression of STING in cartilage samples. For chondrocyte culture, the cartilage tissue was sliced with a blade and digested with 2 mg/ml of type II collagenase (Sigma-Aldrich, St Louis, USA) in Dulbecco’s modified Eagle’s medium (DMEM/F12; Gibco, Grand Island, NY, USA), then incubation for 4 h performed at 37 °C. After rinsing with PBS, the chondrocytes were resuspended and grown in DMEM/F12 with 10% fetal bovine serum and 1% antibiotic and incubated under 5% CO_2_ environment at 37 °C. We employed the passage 0 to evaluate the expression of STING protein in the normal and osteoarthritic cartilage.

### Mouse primary chondrocytes culture

Eight 7-day postnatal mice (C57B6, four males and four females) were anaesthetized to death using phenobarbital. Under aseptic conditions, the articular cartilages were carefully aseptically dissected, collected by microscopic instruments, and were incubated with 1% of type II collagenase (2 mg/ml) for 4 hours at 37 °C. Then, the digested cartilages were resuspended and inoculated in Petri dishes with DMEM/F12 with 10% fetal bovine serum, as well as 1% streptomycin/penicillin antibiotics. The incubator was maintained at a humidified atmosphere and 5% CO2 at 37 °C. Thereafter, the medium was replaced every 2 or 3 days. When cells grew to 80–90% density, they were digested with 0.25% trypsin–ethylenediaminetetraacetic acid solution, and then, transferred onto 10-cm-growth plates at a certain density. In the P0 to P2 passage, there was no significant change in cell morphology; hence, the second passage chondrocytes were utilized for all the in vitro assays.

### Chondrocyte treatment

To explore the expression level of the cGAS-STING signal in mouse chondrocytes, chondrocytes exposed to different quantities of IL-1β (0, 10, 20, 30 as well as 40 ng/mL) for 24 h and with equivalent concentration of IL-1β (10 µM) for different time (0, 6, 12, 24 h). In the functional study of STING in vitro, the chondrocytes were pre-exposed to lv-STING or sh-STING followed IL-1β (10 ng/ml) for 24 h. During the involvement of NF-κB in STING-mediated OA in chondrocyte study, chondrocytes were pretreated with lv-STING or sh-STING or si-P65 followed IL-1β (10 ng/ml) for 24 h.

### Lentivirus transfection

The lenti-STING and lenti-sh-STING were purchased from GeneChem (Shanghai, China). Here, we transfected the Cells at confluence of 30–50%. After 12 h, >95% of cells were viable. In addition, the culture medium was replaced and cultured after 3 days for the subsequent experiment. The transfection efficacy was assessed by western blot.

### siRNA transfection

The si-P65 were acquired from Invitrogen (Carlsbad, CA, USA). Cells were inoculated and cultured in six-well plates for 24 h to achieve a density of 60–70%. Then, 50 nM of control or siRNA duplexes transfection cells were added with the Lipofectamine 2000 siRNA transfection system (Thermo Fisher, UT, USA). Following further treatment, these cells were used for the western blot assays.

### SA-β-gal staining

The senescence of cells was assessed using the senescence-associated-β-GalactoSidase (SA-β-gal) staining kit (Beyotime, Shanghai, China). The senescent chondrocytes are stained blue to indicate the higher SA-β-gal activity.

### Western blot assessment

The total proteins were isolated from the chondrocytes and isolated using the radioimmunoprecipitation assay buffer lysis buffer containing 1 mM of phenylmethanesulfonyl fluoride. The mixture was placed on ice for 10 min, and then centrifugation conducted at 12,000 rpm at 4 °C for 15 min. Afterwards, the concentration of proteins was determined by the BCA protein assay kit (Beyotime). After that, 40 ng of protein was resolved by sodium dodecyl sulfate-polyacrylamide gel electrophoresis and transfer-embedded onto a polyvinylidene difluoride membrane (BioRad, USA). Thereafter, blocking with 5% non-fat milk was performed for 2 hours, subsequently, overnight incubation of the membranes with the primary antibody overnight at 4 °C was done, followed by incubation with the corresponding secondary antibodies for two hours at room temperature. The membranes were then rinsed using Tris-Buffered Saline and Tween 20 thrice and the electrochemiluminescence plus reagent (Invitrogen) employed in visualization. Thereafter, the quantification of the intensities of the blots conducted using the Image Lab 3.0 software (BioRad).

### Immunofluorescence

The chondrocytes were planted on a six-well plate, washed with PBS. They were then fixed using 4% paraformaldehyde, treated in 0.1% Triton X-100 for 15 min, then rinsed with PBS. Then, blocking of the cells was performed using 5% bovine serum for 1 hour, washed by PBS, followed by incubation with the primary antibody at 4 °C overnight. The cells were rinsed again with PBS before incubating them with the fluorescein-labeled goat anti-rabbit IgG antibody (Alexa Fluor 488 or Alexa Fluor 594) (1:500) for 1 hour at room temperature. After that, the cells were rinsed with PBS and stained with 4′,6-diamidino-2-phenylindole (Invitrogen). The images of each segment of cells were captured randomly on a microscope (Olympus Inc., Tokyo, Japan). All codomain computations were performed in five separate experiments, each with 50 cells. The images were demonstrated using Adobe Photoshop 6.0.

For the in vivo studies, tissues were stained by the fluorescent immunostaining technique. Dehydration of the tissues was conducted, and then paraffin-embedded, and sectioned into 5-µm sagittal segments. For the immunofluorescence, the slices were dewaxed with xylene and hydrated with ethanol. Incubation of the segments with 10% bovine serum albumin for 1 hour was done at room temperature in PBS containing Triton X-100. Afterwards, overnight incubation with the primary antibodies at 4 °C in PBS was conducted. Subsequently, the tissues were washed with PBS thrice, then incubated with the goat anti-rabbit secondary antibody for 1 hour at room temperature. After that, the tissues were rinsed with PBS three times, followed by 10 min-incubation. They were then washed again in PBS and then sealed with a coverslip. The microscopic photographs were captured using a fluorescence microscope (Olympus Inc., Tokyo, Japan). Five mice per group were utilized only for quantitative assessment.

### Animal model

Sixty 12-week-old C57BL/6 male wildtype (WT) mice were acquired from the Animal Center of the Chinese Academy of Sciences Shanghai, China. The selection of sample size for animal experiments is carried out as per the preliminary experiments as well as similar well-designed experiments. The investigators were blind to the treatment group during data collection, as well as subsequent data synthesis. OA was induced in experimental mice by the surgical DMM techniques, as previously described^47^. In brief, we anesthetized the mice by intraperitoneal injection of 2% (w/v) pentobarbital (40 mg/kg bw). After that, the capsule was cut medially to the patella tendon, and the meniscus and medial meniscus ligaments were cut using microsurgical scissors. The left knee joints of mice also received arthrotomy, and the medial meniscus ligaments were not excised, thus were employed as the sham group. After surgery, these mice were randomly grouped into four groups: Sham + sh-NC, Sham + sh-STING, DMM + sh-NC, and DMM + sh-STING groups. At 0, 15, 30, and 45 following post-OA surgery, the 10 vl of lentivirus was injected into the articular cavity through the trans-patella tendon approach. The Sham + lv-NC, as well as DMM + lv-NC groups received 10 μL of sh-NC, while the Sham + sh-STING and DMM + sh-STING group were injected with sh-STING. At 8 weeks after the operation, we anesthetized the experimental mice, and then sacrificed, and their knee joints were dissected and processed for histological evaluation.

### Histopathologic analysis

The processed joint tissues, embedded on microscope glass slides, then staining by Safranin O-fast green (SO) dye performed, and a group of experienced histopathologists blindly evaluated the cellular structure and morphology of the cartilage and subchondral bone. The medial femoral condyle along with the medial tibial plateau were evaluated according to OARSI^48^. The synovitis severity was graded following a scoring system, as described previously^49^. The histological scores were assigned to 15 mice in each group.

### Immunohistochemical analysis

The paraffin-embedded sections were dewaxed, hydrated, followed by blocking with 3% hydrogen peroxide endogenous peroxidase. We then incubated the segments with 0.4% pepsin (Sangon Biotech, Shanghai, China) in 5 mM of HCl at 37 °C for 20 min for antigen retrieval. Afterwards, incubation of the segments using 5% bovine serum albumin was accomplished at room temperature for 30 min, and incubation with the primary antibody performed overnight at 4 °C. The blinded observers quantified the rate of positive cells in each section. Five mice from each group were employed for the quantitative assessment.

### Statistical analysis

Statistical analyses were carried out in the SPSS statistical software program 20.0 (IBM, Armonk, NY, USA). The data were analyzed by one-way analysis of variance, followed by Tukey’s test, in order to compare the control and treatment groups. The nonparametric data (OARSI grading and synovitis scores) were analyzed using the Kruskal–Wallis *H* test. A *P* value of <0.05 indicated statistical significance.

### Ethics statement

The human articular cartilage tissue collection and human cartilage tissue-related experiments were approved by Ethical Committee of the Second Affiliated Hospital, Wenzhou Medical University (ethic code: LCKY2019-57) and following the guidelines of the Declaration of Helsinki. All surgical interventions, treatments, and postoperative animal care procedures are strictly implemented following the “Guidelines for the Care and Use of Laboratory Animals of the National Institutes of Health” and are approved by the Animal Care and Use Committee of Wenzhou Medical University (ethic code: wydw2019- 0027).

## Supplementary information

figS1

Figure legends figS1
