# Supplementary figures and images for: STING promotes senescence, apoptosis, and extracellular matrix degradation in osteoarthritis via the NF-κB signaling pathway
Source: Cell Death Dis. 2021 Jan 4;12(1):13. doi: 10.1038/s41419-020-03341-9 (PMC7791051; doi:10.1038/s41419-020-03341-9)

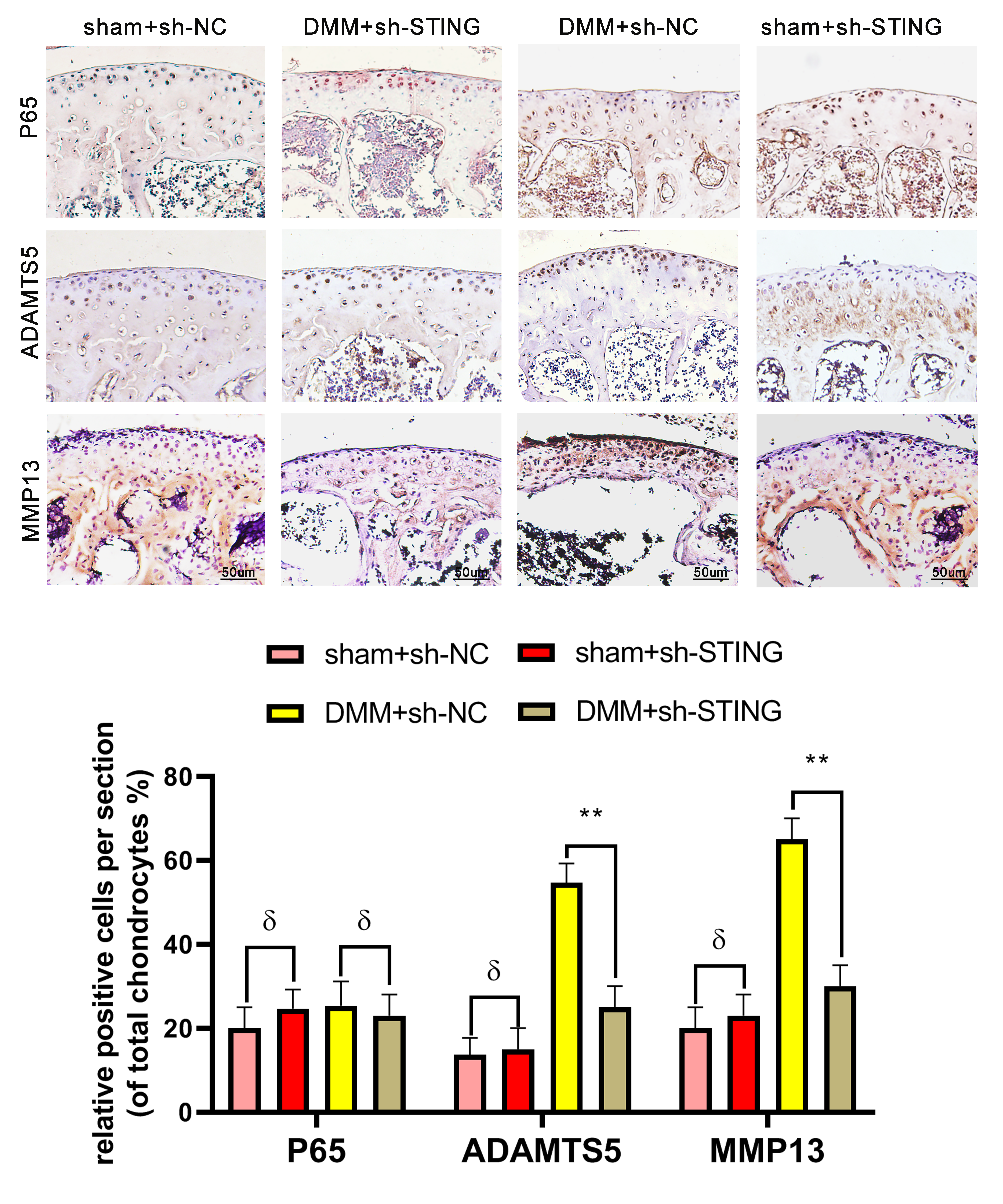

Supplement: Supplementary file 1 — figS1 [file 41419_2020_3341_MOESM1_ESM.tif]
