# Supplementary material for: STING promotes senescence, apoptosis, and extracellular matrix degradation in osteoarthritis via the NF-κB signaling pathway
Source: Cell Death Dis. 2021 Jan 4;12(1):13. doi: 10.1038/s41419-020-03341-9 (PMC7791051; doi:10.1038/s41419-020-03341-9)
Supplement: Supplementary file 2 — Figure legends figS1 [file 41419_2020_3341_MOESM2_ESM.docx]

**Figure S1 The expression of STING, P65, MMP13, ADAMTS5 expression in mouse cartilage**

(**A-B**) The expression of P65, AMADTS5, MMP13 explored by immunohistochemistry in mouse cartilage (bar: 50μm). All data were indicated as mean ± SD (n = 15); **P<0.01.
